# Selective Laser Melting Produced Ti-6Al-4V: Post-Process Heat Treatments to Achieve Superior Tensile Properties

**DOI:** 10.3390/ma11010146

**Published:** 2018-01-17

**Authors:** Gerrit M. Ter Haar, Thorsten H. Becker

**Affiliations:** Materials Engineering Group, Department of Mechanical & Mechatronic Engineering, University of Stellenbosch, Stellenbosch 7600, South Africa; tbecker@sun.ac.za

**Keywords:** additive manufacturing, powder bed fusion, selective laser melting, heat treatment, titanium alloys, phase transformation, tensile properties

## Abstract

Current post-process heat treatments applied to selective laser melting produced Ti-6Al-4V do not achieve the same microstructure and therefore superior tensile behaviour of thermomechanical processed wrought Ti-6Al-4V. Due to the growing demand for selective laser melting produced parts in industry, research and development towards improved mechanical properties is ongoing. This study is aimed at developing post-process annealing strategies to improve tensile behaviour of selective laser melting produced Ti-6Al-4V parts. Optical and electron microscopy was used to study α grain morphology as a function of annealing temperature, hold time and cooling rate. Quasi-static uniaxial tensile tests were used to measure tensile behaviour of different annealed parts. It was found that elongated α’/α grains can be fragmented into equiaxial grains through applying a high temperature annealing strategy. It is shown that bi-modal microstructures achieve a superior tensile ductility to current heat treated selective laser melting produced Ti-6Al-4V samples.

## 1. Introduction

Since the emergence of selective laser melting (SLM) as a humble rapid prototyping technology in 2006, the process has advanced exponentially to a point where it has become the most broadly used powder-bed fusion manufacturing process in industry [1]. SLM produced titanium alloys have been used for aircraft components and customised medical implants [2]; the ability to produce complex part geometries, reduce manufacturing lead time and reduce material waste has made SLM produced titanium a competitive alternative to conventional manufacturing processes. Considerable attention has been directed toward Ti-6Al-4V, a 6% aluminium, 4% vanadium titanium alloy that is considered the ‘workhorse’ in the titanium industry due to its excellent material properties.

SLM produced Ti-6Al-4V parts in an as-built state are unable to achieve the high material performance of its wrought counterparts [3,4]. While parts are stronger (with regards to its ultimate tensile strength of up to ~1200 MPa [5]), ductility and toughness are low (with an elongation to failure reported at less than 5% in some cases [5]). Low ductility is attributed to the presence of an undesired α’ (martensite) microstructure within long columnar prior β (beta) grains [6,7,8].

Microstructural morphology is the fundamental fingerprint for mechanical behaviour. In Ti-6Al-4V, the grain morphology (lamella vs. equiaxed), grain size (fine vs. coarse) and the two phase α + β (alpha + beta) structure prescribe the plastic deformation behaviour of the material [9]. Wrought Ti-6Al-4V is thermomechanically processed to achieve a bi-modal microstructure that consists of large equiaxed/globular α grains in a matrix of elongated α + β lamellar grains. This is referred to as primary and secondary α due to the order of formation in a duplex annealing process and allows for a balance of the advantages offered by a fine lamellar vs. an equiaxed microstructure (i.e., strength vs. ductility).

Bi-modal microstructures are achieved through a thermomechanical process (TMP) that allows for grain fragmentation and globularisation [10]. The microstructural morphology is first tailored using hot working to induce dislocations that fragment α grains followed by fast cooling (air cooling or water quench) from a solid solution temperature (SST) (between 900 and 970 °C according to Aerospace Material Specification H-81200C and findings by Semiatin et al. [11]). Equiaxial α grains form because of preferential globularisation at β grain boundaries. Subsequently, a second anneal is used to relieve residual stresses and/or decomposition of α’ into a dual α + β lamellar phase.

The challenge of achieving a bi-modal microstructure through post-processing in SLM produced Ti-6Al-4V is that the starting microstructure is martensitic and the SLM technology aims for near net shape part production and therefore parts cannot be TMPed. Research studies aimed at improving part ductility have shown that annealing is effective in decomposing α’ into a stable dual phase lamellar α + β microstructure [5,7,12,13,14,15,16,17]. However, the annealing strategies that are applied to wrought samples do not have the same effect on SLM produced Ti-6Al-4V; the α’ forms into a coarse elongated lamella morphology. This improves ductility, however at the cost of strength [5,7,12,13,14,15,16,17]. While this improvement in ductility is satisfactory according to ASTM standards (such as ASTM F2924–14 [18]), ductility vs. strength is still significantly less than that of the wrought material [9,19].

Potential lies in optimising mechanical properties of SLM produced Ti-6Al-4V. Annealing strategies to optimise mechanical properties need to be developed that explain grain morphological transformation as a function of annealing temperature and holding time in the context of developing a superior microstructure, i.e., a bi-modal microstructure that balances advantages of a fine lamellar vs. equiaxed microstructure.

This study aims to explain grain morphological transformation as a function of annealing temperature, holding time and cooling rate of SLM produced Ti-6Al-4V to contribute to the understanding of the mechanism of grain morphology transformation in the solid solution temperature region (SSTR). This allows for heat treatment strategies, aimed at achieving a bi-modal microstructure, to be tailored for the specific case in which the starting microstructure is martensitic. Annealing strategies are conducted in the low-, medium- and high-SSTR. While the low and medium are conducted to determine the annealing strategy for decomposing α’ into a stable dual phase lamellar α + β microstructure, the high is aimed at understanding fragmentation and globularisation to achieve more equiaxed primary α-grains.

The scope of the study focuses on improving strength and ductility in order to achieve tensile properties that are comparable to its wrought counterpart. It is illustrated, for the first time, that achieving a bi-modal microstructure in SLM produced Ti-6Al-4V is possible and that a superior microstructure to current annealing strategies below the β-transus temperature is possible.

## 2. Materials and Methods

### 2.1. Powder

**Figure 1:**
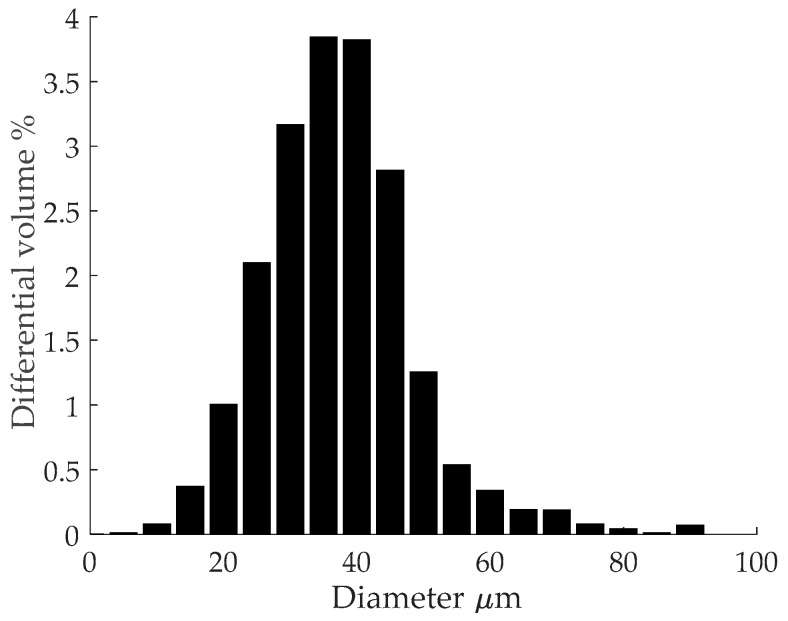
Powder size distribution.

Ti-6Al-4V ELI (Grade 23) atomised spherical powder was acquired from TLS Technik GmbH & Co. (Bitterfeld-Wolfen, Germany). Collaborators in the study performed elemental and particle size analysis of the powder. Results of elemental composition and particle size distribution were first published by Thejane et al. [20]. Powder size distribution was measured using a *MICROTRAC* SI/S3500 laser scanner (Microtrac GmbH, Krefeld, Germany). The results are plotted in Figure 1 and they show that the powder size distribution agrees with the manufacturer’s specifications. Powder elemental analysis was done by Inductively Coupled Plasma Optical Emissions Spectroscopy (ICP-EOS) to determine metallic composition. Inert gas fusion was used to measure the composition of gas elements in the powder. ICP-EOS was done using a SPECTRO ARCOS machine (SPECTRO Analytical Instruments GmbH, Kleve, Germany) while inert gas fusion was done using an ELTRA OHN 2000 (ELTRA GmbH, Haan, Germany). The results of elemental analysis on the virgin powder are given in Table 1. This data shows that the alloy elements in the powder are within the required standard set by ASTM F1580-12 [21]. The oxygen concentration in the powder (weight %) after three consecutive builds was measured to be: 0.082, 0.092 and 0.096. Although slight oxygen pick-up is recorded after each build; after three builds the oxygen concentration is still below the 0.13% recommended by the standard.

### 2.2. Build Parameters and Scan Strategy

**Figure 2:**
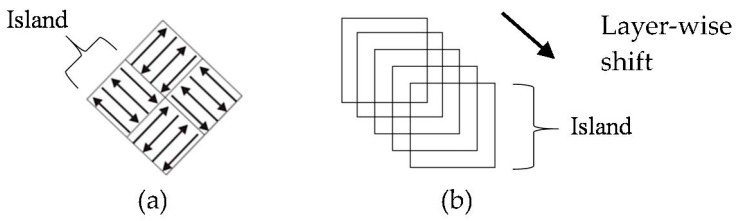
(**a**) island hatch strategy [22] and (**b**) island shift strategy.

All samples were built using a LaserCUSING *M2* machine by Concept Laser GmbH (Lichtenfels, Germany). Samples were deposited on a wrought Ti-6Al-4V substrate in an argon environment with an O_2_ concentration below 50 ppm. Scan parameters are tabulated in Table 2. The scan strategy used was Concept Laser’s patented ‘island’ scan strategy in which each layer is divided into 5 mm × 5 mm square islands and exposed in a zig-zag fashion. Scan vectors in each island are exposed at 90° with respect to the neighbouring island and alternating layers. A shift of 1 mm with respect to the previous island layer is used to limit porosity build-up. Figure 2a illustrates the exposure strategy and Figure 2b the shift movement of a single island over five consecutive layers.

### 2.3. Samples and Testing

Thirty cylindrical samples of dimensions 15 mm in diameter and 93 mm in length were built for tensile tests while 20 short samples of 20 mm of the same diameter where built for microstructural analysis. All samples were built in the *z*-orientation, i.e., with their longitudinal axis parallel to the build plane. After the respective heat treatment, long samples were machined into a cylindrical ‘dog bone’ shape with a gauge length (G) of 25 mm and a gauge diameter (D) of 5 mm according to 5-to-1 ratio stipulated by ASTM E8M–15 [23]. Finally, the gauge section was polished to a mirror finish. Tests were conducted using the MTS Criterion 44. All tests were displacement controlled with a strain rate of 0.001 s^−1^. Elongation to failure ($\varepsilon_{f}$) was measured by putting the two broken halves together and measuring the gauge length after failure (G_f_) as advised by ASTM E8M–15 [23]. Therefore, $\varepsilon_{f}$ = (G_f_ − G)/G.

### 2.4. Density Measurements

Sample density was measured, and porosity morphology was observed to determine to what extent porosity could affect tensile results. Porosity investigations were undertaken using the Archimedes principle according to ASTM B311–13 [24], complimented by X-ray Computed Tomography (XCT). For Archimedes measurements, a Kern & Sohn GmbH (Balingen, Germany), model ABT 120-5DM scale was used. The suspension liquid used was isopropanol instead of water to reduce the formation of air bubbles on the rough surface of the samples and thereby achieve more consistent measurements. For XCT measurements, a General Electric Phoenix V|Tome|X L240 (Boston, MA, USA) was used. The middle gauge section of the samples was scanned over a region of approximately 5 mm × 5 mm allowing for a voxel resolution of 3 µm^3^.

### 2.5. Heat Treatments

The heat treatments were undertaken before machining of the samples using a 5 kW Gallenkamp muffle furnaces (Weiss Technik, Heuchelheim, Germany) and a EUROTHERM temperature controller (Schneider Electric, Worthing, United Kingdom). Sample temperature was measured by inserting a Type-K thermocouple into the furnace and probing the sample being annealed. Dwell times and cooling rates were heat treatment specific. A muffle furnace was chosen to allow for furnace cooling (FC), air cooling (AC) and water quenching (WQ). Oxidation effects such as scale and α-case hardening were removed through machining afterwards.

### 2.6. Microstructural Analysis

Metallographic samples were ground and polished using Buehler metallography equipment. Light Optical Microscopy (LOM) was undertaken using an Olympus GX51 optical microscope (Olympus Corporation, Tokyo , Japan) and *Stream Essentials* (ver. 1.9.1.) on etched samples using Kroll’s Reagent (92 vol % H_2_O, 6 vol % HNO_3_ and 2 vol % HF) for 5 to 10 s to reveal the microstructure. For electron microscopy analysis, a Zeiss *MERLIN* scanning electron microscope (SEM) (Zeiss, Oberkochen, Germany) in conjunction with a backscatter detector (BSD) was used.

Individual grain width measurements were done with Stream Essentials software tools. Volumetric α/β phase fractions were assumed to coincide with the area phase fractions of a 2D micrograph. Minimum and maximum grain widths were measured as mean values using the line-intercept method automated in Matlab 2017a through the help of Matlab’s Image Processing Toolbox. 

X-ray diffraction was done using a Bruker D2 Phaser diffractometer (Cu Kα source) with a standard Bragg Brentano geometry. Accusation dwell time was 0.75 s at steps of 0.01°.

## 3. Heat Treatment Design and Approach

**Figure 3:**
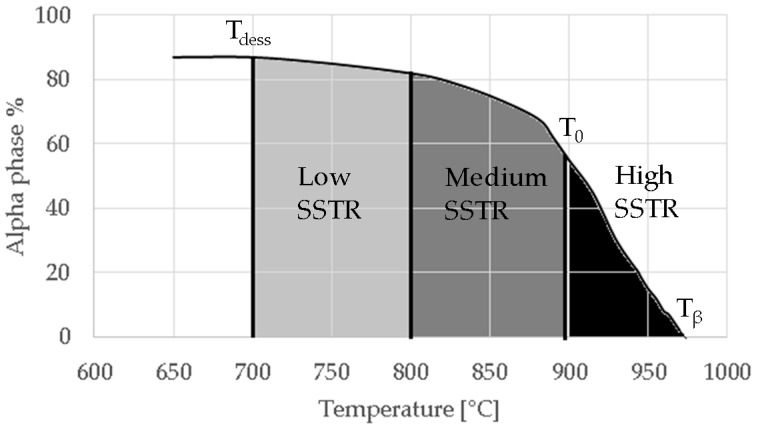
Schematic of temperature regions and key temperatures in the SSTR vs estimated α phase fraction.

The temperature region chosen for annealing in this study was within the SSTR, illustrated in Figure 3. In this solid solution, the α-phase is the solute and the β phase the matrix. In this study, the SSTR was defined as the temperature region between the dissolution temperature (T_dess_) and the β-transus temperature (T_β_). The dissolution temperature (chosen as a single temperature point) is the temperature region where the β phase starts to exponentially increase in volume percentage as the α-phase dissolves into β-phase. This temperature has been defined by Kelly as 705 °C [21] which was also used in this study. 

The SSTR was furthermore divided into low-, medium- and high-SSTR regions. The low- and medium-SSTRs lie below the critical temperature (T_0_) and the high-SSTR lies above T_0_. The critical temperature is defined as the temperature above which α’ forms upon fast cooling (water—WQ or air cooling—AC). This temperature has been calculated based on a thermodynamic database by Lu et al. [25] and Ji et al. [26] who respectively found T_0_ to be 893 °C and 872 °C. The low- and medium-SSTR is separated by the temperature region considered by various authors to be where α’ decomposes into α upon heating and has been shown to provide good tensile results [7,12,14]. 

The heat treatments tabulated in Table 3 were designed to accentuate the effects of dwell temperature, dwell time and cooling rate. Since α grain growth is limited in low- and medium-SSTR heat treatments, variations in air and furnace cooling as a function of dwell time were considered. High-SSTR heat treatments used WQ to limit α grain growth, to best show the extent of α grain growth and fragmentation. As such, a α/β phase fraction is not obtainable and a α/α’ phase fraction is given that is representative of a α/β phase fraction.

## 4. Results

### 4.1. Microstructure

#### 4.1.1. As-Built Samples

**Figure 4:**
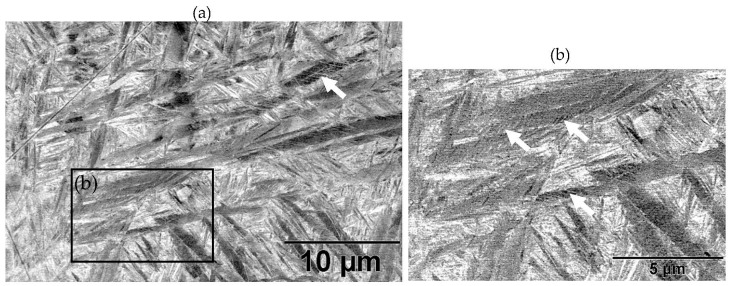
As-built microstructure: (**a**) BSD-SEM image revealing fine tertiary and quadric α’ grains and sub-grain twinning (indicated with arrow); and (**b**) an enlarge image showing sub-grain twinning (indicated with arrows).

Figure 4 shows the as-built microstructure. As expected, the microstructure consists of a fine single phase α’ needle-like grain structure. The measured range of grain sizes was found to be extremely large (between 10 nm and 3 µm). A hierarchical structure (similar to that measured by Yang et al. [27]) was observed which consists of primary, secondary, ternary and quartic α’ grains. Table 4 lists the measured lengths of the major and minor axis of α’ grains.

The BSD micrograph in Figure 4 shows the fineness of the ternary and quartic structure. This hierarchical structure forms naturally in α’, but gets accentuated by the cyclic heating and cooling in the SLM process [27]. The microstructure’s hierarchical structure and grain size distribution is a key characteristic influencing the microstructural morphology transformation, as discussed later in this paper. Twinning dislocations can be found, indicated by arrows. This internal twinning has been observed numerous times in the SLM induced α’ phase [8,27,28,29,30].

#### 4.1.2. Samples Annealed in the Low- and Medium-SSTRs

Table 5 provides LOM micrographs and measured grain sizes of samples annealed at low- and medium-SSTRs. The dark regions indicate etched β phase.

Annealing in the low-SSTR shows transformation from α’ to lamella α + β phases. Table 5 shows an increase of the fine as-built microstructure to a 0.5 μm minimum lamella grain width. It is observed that quartic α’ grains have been dissolved with little primary, secondary and ternary α grain growth. The measured increase in the minimum grain size and insignificant growth in maximum grain size suggests that the fine quartic α’ grains have transformed to β, whereas primary, secondary and ternary α’ grains have transformed to α with minimal grain growth. 

Annealing in the medium-SSTR shows grain growth of primary, secondary and ternary grain indicated by the in the maximum grain sizes given in Table 5. Significant grain growth is measured when comparing cooling rates where furnace cooling (FC) allowed for a comparatively large amount of α grain growth within a shorter time period. β grain width was found to be thinner and the α/β phase fraction was measured to be larger in FC samples, compared to air cooled (AC) samples. These α/β phase fractions agree with those published in open literature [11,31,32,33,34].

#### 4.1.3. Samples Annealed in the High-SSTR

Annealing in the high-SSTR shows a microstructure that mainly consists of transformed primary α’ grains into lamella α in a β matrix, where tertiary and quadric α’ grains have transformed to β. This suggests that the hierarchical martensitic rank favours higher order phases based on their relative size with complete transformation occurring at the β-transus temperature.

Table 6 summarises microstructure results of samples annealed at 910 °C for various dwell times, followed by water quenching (WQ). The light regions indicate β phase. By comparing α/α’ phase fraction and therefore the α/β phase fraction at quench temperature, it becomes apparent that 30 min dwell time is not sufficient for phase transformation to reach a steady state equilibrium. Phase fraction equilibrium (41% α phase) is reached between 30 min and 2 h. Grain growth is almost insignificant during this time, however, after 8 h a noticeable increase in grain width is measured. The micrograph of the sample annealed at 945 °C shows a larger amount of grain growth for 4 h dwell time. α grain fragmentation is evident (indicated by solid arrow) at prior β grain boundaries and triple points resulting in grain globularisation (indicated by hollow arrows). The given phase fractions agree with the equilibrium phase fraction given by open literature [11,31,32,33,34].

Table 7 summarises the microstructure of samples annealed at 960 °C. Similarly, a dwell time of 30 min does not allow for an equilibrium in the phase percentage. It is interesting to note that the α/α’phase fraction and grain size is similar to that of the sample annealed at 910 °C for 30 min. After 4 h significant grain fragmentation of elongated α grains was observed (indicated by solid arrows) resulting in globularisation (indicated by hollow arrows). Furnace cooling from 960 °C after 4 h causes significant growth in grain width. Water quenching at 930 and 900 °C produced thick primary α grains in a matrix of α’. These α grains are no longer elongated and essentially become equiaxed due to the reduction in aspect ratio. The phase fractions agree with equilibrium phase fraction at these temperatures presented in open literature [11,31,32,33,34].

### 4.2. Duplex Anneal

Fast cooling by WQ from above T_0_ achieves a bi-modal microstructure of α in a matrix of α’. The α’ is an unwanted phase due to its metastable and brittle nature. Thus, a second annealing step is introduced to decompose the newly formed *α*’ into (*α* + β) lamellar using a low-SSTR anneal.

Table 8 summarises a bi-modal microstructure achieved through such a duplex annealing strategy. Shown is a bi-modal microstructure consisting of a equiaxed primary α phase and lamella secondary α. Grain sizes vary from a relatively large 5 μm globular structure to an elongated 1 μm grain width. 

### 4.3. Tensile Behaviour

**Figure 5:**
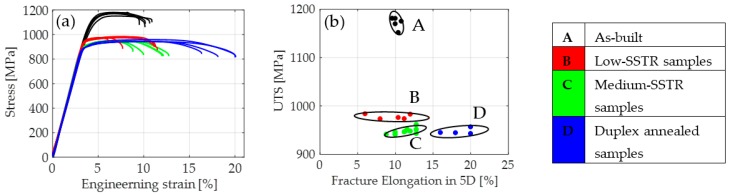
Plot of (**a**) tensile stress-strain curves and (**b**) ultimate tensile strength (UTS) vs fracture elongation for the four groups of tensile samples.

The mean and median sample densities were measured at 99.25% and 99.22% respectively while the minimum and maximum density of the build was measured at 98.78% and 99.85% respectively. Using XCT, the geometry of porosity was observed to be spherical. The results of uniaxial tensile tests are presented in a plot of UTS vs. % elongation shown in Figure 5. Samples in the plot are grouped with circles which represent 1.5 standard deviations of the group’s mean.

Ductility of the as-built group was found to lie in the upper-end of reported values (5–10%) [35]. This could be attributed to the presence of compressive residual stresses in an as-built state that has been shown to act as a strengthening mechanism by inhibiting crack nucleation and growth [36]. Low- and medium-SSTR samples both achieved a varied improvement in ductility at a significant cost of strength. Duplex samples achieved a noteworthy increase in ductility with a strength similar to samples annealed in the medium-SSTR. Ductility of low-SSTR samples was on average lower than both the as-built and medium-SSTR samples due to two samples performing poorly. 

Mechanical properties of groups A to C agree well with that of literature [5,7,12,13,14,15,16,17]. Results obtained for group D agree with ductility achieved by solid solution heat treatments on wrought Ti-6Al-4V [19].

## 5. Discussion

### 5.1. Background to Morphological Transformations

Two fundamental mechanisms that drive morphological transformations have previously been observed: (i) Phase transformation is driven by the minimisation of Gibbs free energy [26,37,38,39]. The major contribution being the entropy or chemical potential energy of alloying elementals in the respective α and β phases [26]. A change in α/β phase fraction through phase transformation occurs as nucleation and growth of the phase until a stable/equilibrium alloy concentration is reached [40]. This is attained through atomic diffusion across the phase boundary. The diffusion rate (and therefore the phase transformation rate) is complexly dependent on a large range of parameters including temperature, interface mobility, diffusivity and the fluctuating alloy concentration across the phase [40]. Upon cooling, the mechanism is sensitive to the rate of change in temperature, where the phase transformation route of least energy is taken favouring atomic diffusion during furnace cooling and shear displacement transformation during fast cooling, the later resulting in the formation of α’ [26]. (ii) Grain morphology transformations are driven by grain surface-area minimisation; to minimise the total surface energy smaller grains shrink while larger grains grow in size [41]. The mechanism is dependent on time and temperature, and the rate of grain growth increases with an increase in temperature. While both mechanisms occur simultaneously and are competing, the second mechanism becomes more noticeable at higher temperatures.

### 5.2. Low- and Medium-SSTR Heat Treatments

**Figure 6:**
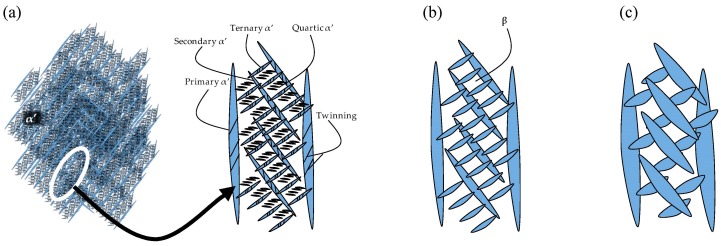
Schematic of (**a**) martensitic α’ hierarchical structure and annealing in the (**b**) low- and (**c**) medium-SSTR.

Annealing below the critical temperature is currently the most common annealing strategy for SLM produced Ti-6Al-4V. At low- and medium-SSTR heat treatments, the nucleation and growth of β-phase at grain boundaries and twinning dislocations start at T*_dess_* and below T_0_. Due to the hierarchical structure of α’, illustrated by Figure 6a, the phase transformation (morphology transformation mechanism (i)) of α’ to β during annealing below T_0_ is initiated by the transformation of the smallest α’ grains first, followed by subsequent larger grains (quartic → ternary → secondary). Figure 6b,c demonstrate this mechanism by showing β-phase replacing the quartic α’grains. Since primary α’ grains are largest, a complete transformation of these to β occur last at T_β_. As such, primary α’ → α transformed grains become more dominant in samples annealed at increasingly higher temperatures. This was observed by an increase in the measured minimum grain width of sub-T_0_ treated samples. Thus, morphology transformation mechanisms (i) can be argued to be more dominant.

The rate of α grain growth at isothermal temperature was measured to be low, increasing at higher temperatures. At low- to medium-SSTR heat treatments, primary and secondary α-phase growth was in the order of 1 µm. Morphology transformation mechanism (i) is also argued to be dominant during an isothermal hold. The dominant mechanism influencing grain growth at this temperature range was shown to be the cooling rate, where furnace cooling allows for further α grain growth by 1 to 2 µm and an increase in α phase percentage.

**Figure 7:**
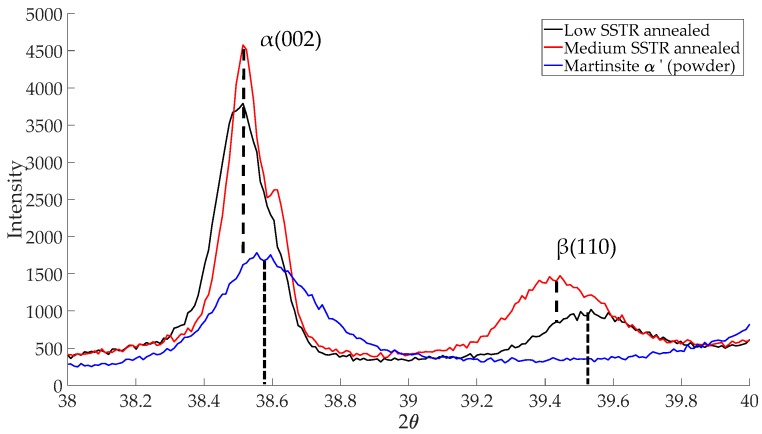
XRD plot of martensitic powder and samples annealed in the low and medium-SSTR.

Figure 7 depicts Bragg peaks between 2θ angles of 38° to 40° for low-, medium-SSTR annealed and powder samples. Since both the as-built sample and the power consist of α’ phase, but the power is considered to be stress-free, the powder was used as a reference α’ phase material for XRD analysis. Both low- and medium-SSTR annealed samples show α-phase (hexagonal close-pack, HCP, lattice structure) Bragg peak positions that are aligned with one another. The peak position of the powder to the right of the annealed samples peak is due to α’-HCP lattice spacing being smaller than that of α-HCP. This is due to a higher concentration of alloying elements in the powder’s martensitic phase [42]. Aluminium and vanadium both have smaller atomic radii, 0.143 nm and 0.132 nm respectively, than titanium, 0.147 nm [43]. Decomposition α’ to α is observed by the peak shift of the annealed samples to the right indicating a larger lattice spacing and therefore a lower alloying concentration.

During low-SSTR heat treatments, smaller quartic and ternary grain transformation is driven by diffusion (mechanism (i)) whereby smaller α’ grains shrink and dissolve to form β. This is visible in Figure 7 by the formation of the β-phase (body centred cubic, BCC, lattice structure) Bragg peak. At medium-SSTR, the β-phase peak shift indicates higher concentrations of vanadium (low-SSTR samples have a smaller β-phase lattice spacing). Furthermore, the greater β-phase peak intensity of the medium-SSTR sample is indicative of a higher volume percentage of β-phase in this sample. The observed morphological change in microstructure is therefore seen to be driven by a combination of the diffusion (mechanism (i)) and surface energy minimisation (mechanism (ii)).

Furthermore, due to the reduction in β lattice constant, a larger mismatch of the α/β lattice constants exists at the phase interfaces. This mismatch promotes the formation of dislocations once deformation begins [42] and therefore results in a higher interface strength and hence increased hardness and yield strength. Furthermore, a widening of α/β interface will weaken interface strength while a narrower interface will increase strength [42].

The tensile behaviour of the low- and medium-SSTR annealed samples reveal two important considerations: (i) the medium-SSTR annealed samples achieved a lower strength compared to the low-SSTR annealed samples. This is due to a widening of the lamella α laths and a larger β lattice spacing allowing for a lesser degree of mismatch between HCP and BCC slip planes. (ii) the ductility of the low- and medium-SSTR annealed samples are similar. Therefore, it would be advised to anneal in the low-SSTR to achieve a superior strength.

### 5.3. High-SSTR and Duplex Heat Treatments

**Figure 8:**
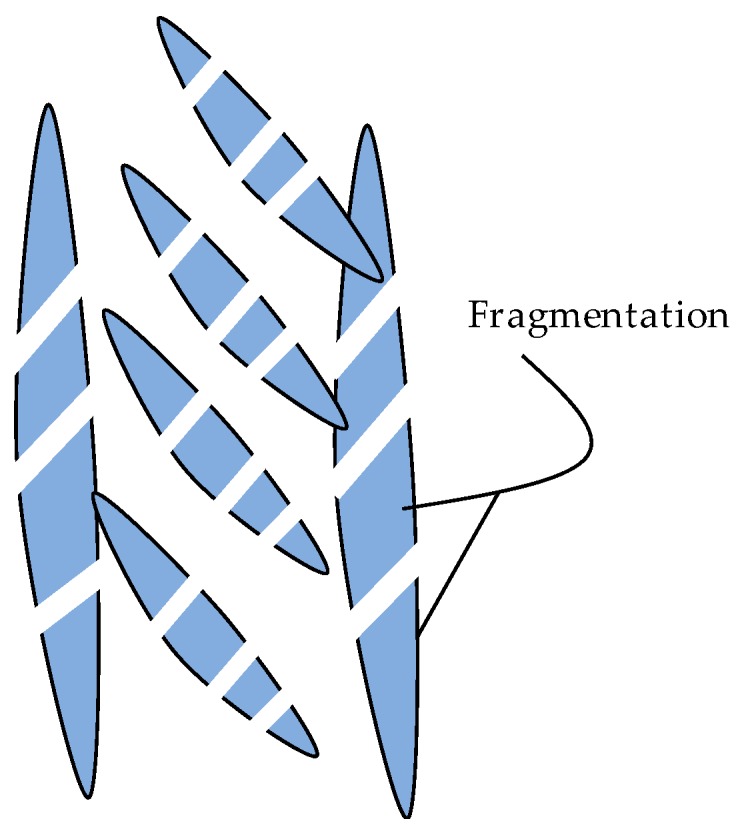
Schematic of morphology transformation during annealing in the high-SSTR.

Figure 8 depicts the morphology of samples in the high-SSTR. Primary and secondary α grain fragmentation is abundant, identifiable by the formation and widening of twinning sites where β forms. The degree of β formation enhances the fragmentation process and, therefore, grain fragmentation increases significantly with an increase in temperature above *T*_0_. Grain growth is increased after fragmentation by surface energy minimisation (mechanisms (ii)). In addition, the diffusion mechanism (mechanisms (i)) is enhanced at slower cooling rates, resulting in massive α grain growth. At high cooling rates, fragmented primary and secondary α grains are ‘frozen’ in a matrix of newly formed α’. Since the brittle and metastable nature of α’ is not always desired, a second anneal cycle at a low-SSTR is suggested aimed at transforming α’ to α. 

The large size of the fragmented primary α grains greatly improves part ductility. The UTS of bi-modal samples coincided with that of the medium-SSTR samples. It is concluded that the secondary α + β structure has a dominant effect on strength since the addition of larger primary α grains in the bi-modal microstructure did not significantly affect the UTS. A further increase in strength may be obtained by refinement of the secondary α + β structure as argued in Section 5.2.

## 6. Conclusions

This study pursued the improvement in tensile behaviour (UTS vs. ductility) of SLM produced Ti-6Al-4V beyond that which is currently achieved by post-process published annealing strategies. The study was aimed at obtaining a fundamental understanding of morphology transformation of SLM produced Ti-6Al-4V α’ as a function of temperature, hold time and cooling rate in the SSTR. A bi-modal microstructure without the need for thermomechanical working was developed. Through understanding α-grain morphology transformation, the research demonstrated the advantages of internal twinning dislocations inherent in α’ and the use thereof to fragment α grains during a high-SSTR anneal. Bi-modal microstructures, consisting of fragmented equiaxial primary α grains in a matrix of (α + β) lamellar, can be achieved through fast cooling from a high-SSTR followed by a low-SSTR annealing step. The paper showed that a microstructure that achieves superior tensile properties to standard annealing strategies is possible for SLM produced Ti-6Al-4V.

## Figures and Tables

**Table 1:** Powder elemental composition (weight %).

Al	V	Fe	N	O	Ti
6.08	3.88	0.17	0.023	0.090	90

**Table 2:** Printing process parameters.

Power (P) (W)	Velocity (v) (mm/s)	Layer Thickness (t) (μm)	Laser Spot Diameter (d) (μm)	Hatch Spacing (h) (μm)	Energy Density (Ev=P/vth) (J/mm^3^)
100	600	30	150	105	53

**Table 3:** Specifications for heat treatment strategies.

Annealing Strategy	Temperature (°C)	Hold Time (h)	Method of Cooling *
Low-SSTR	750	8	AC
Medium-SSTR	800	2	FC
	870	2	AC
		4	FC
High-SSTR	910	0.5	WQ
		2	WQ
		8	WQ
	945	4	WQ
	960	0.5	WQ
		4	WQ
		4	FC to 930 °C then WQ
		4	FC to 900 °C then WQ
Duplex	910 and 750	8 and 4	WQ and FC

* WQ—Water quench, FC—Furnace cooled, AC—Air cooled.

**Table 4:** Hierarchical structure of α’.

Type of α’	Length of Major Axis	Length of Minor Axis
Primary α’	(>20 μm)	(1–3 μm)
Secondary α’	(10–20 μm)	(100–900 nm)
Ternary α’	(2–10 μm)	(10–90 nm)
Quartic α’	(<2 μm)	(<10 nm)

**Table 5:** Microstructure summary—low and medium-SSTR annealing.

Temperature (°C), Hold Time	LOM Micrograph	α/β Phase Fraction	α Grain Width (Min–Max)
750, 8 h AC	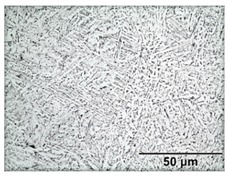	87%	0.5–1.5 μm
800, 2 h, FC	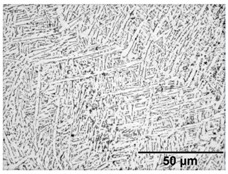	87%	1–2 μm
870, 4 h AC	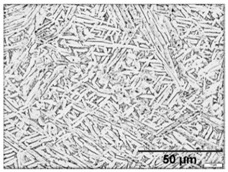	65%	1.5–2.5 μm
870, 2 h, FC	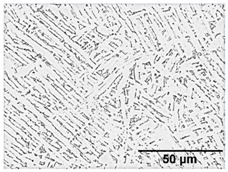	88%	1.5–3.5 μm

**Table 6:** Microstructure—annealing at 910 °C and 945 °C. Grain fragmentation and globularisation is indicated by solid and hollow arrows respectively.

Temperature (°C), Hold Time	BSD-SE Micrograph	α/α’ Phase Fraction	Median α Grain Width
910, 30 min	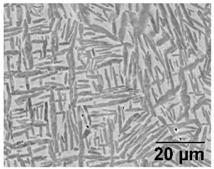	60%	1.5 μm
910, 2 h	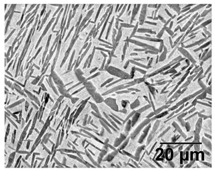	41%	1.5 μm
910, 8 h	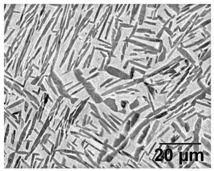	41%	2.8 μm
945, 4 h	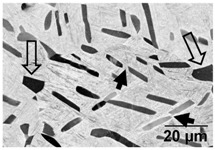	20%	3.5 μm

**Table 7:** Microstructure—annealing at 960 °C.

Temperature (°C), Hold Time	BSD-SE Micrograph	α/α’ Phase Fraction	Median α Grain Width
960, 30 min	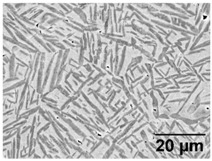	58%	1.5 μm
960, 4 h	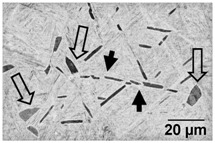	8%	1.5 μm
960, 4 h, FC to 930	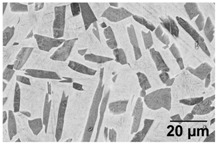	30%	8 μm
960, 4 h, FC to 900	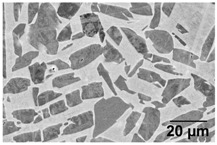	48%	11 μm

**Table 8:** Bi-modal microstructure summary.

Temperature (°C), Hold Time	BSD-SE Micrograph *	α_p_/α_s_ Phase Fraction	Median Grain Width
910, 8 h WQ, followed by 750 4 h FC	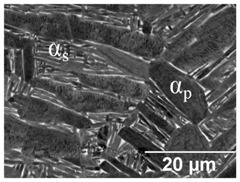	~41%	Primary: 5 μm Secondary: 1 μm

* α_p_—primary α, α_s_—secondary α.
